# Effects of West Nile virus on behavioral and cognitive performance, cortical Aβ pathology, viral loads, and immune measures of middle-aged NL-G-F/E3 and NL-G-F/E4 mice

**DOI:** 10.3389/fnagi.2025.1600119

**Published:** 2025-06-17

**Authors:** Abigail O’Niel, Christopher J. Parkins, Alexandra Pederson, Elizabeth Saltonstall, Emily Bunnell, Ria Aggarwal, Phoebe Sandholm, Kat Kessler, Henry F. Harrison, Jessica L. Smith, Alec J. Hirsch, Jacob Raber

**Affiliations:** ^1^Department of Behavioral Neuroscience, Oregon Health and Science University, Portland, OR, United States; ^2^Vaccine and Gene Therapy Center, Oregon Health and Science University, Portland, OR, United States; ^3^Department of Neurology, Oregon Health and Science University, Portland, OR, United States; ^4^Department of Radiation Medicine, Oregon Health and Science University, Portland, OR, United States; ^5^Division of Neuroscience, ONPRC, Oregon Health and Science University, Portland, OR, United States

**Keywords:** West Nile virus, apolipoprotein E, amyloid precursor protein, behavioral testing, cognitive testing, body temperature, amyloid pathology, viral load

## Abstract

**Introduction:**

West Nile Virus (WNV) can cause severe and long-lasting neurological disease and results in some neuropathology and neuroinflammation seen in Alzheimer’s disease (AD). Exposure to WNV might impact AD-relevant behavioral and cognitive performance and neuropathology via AD-susceptibility genes (i.e., E4) and by inducing neuroinflammation (i.e., increases in TCR-α, IFN-γ, TNF-α, and CXCL- 10). There are three human apolipoprotein E (E) isoforms, which play a role in cholesterol metabolism: E2, E3, and E4. Compared to E3, E4 is an AD risk factor.

**Methods:**

We crossed knock-in (KI) mice expressing human amyloid precursor protein (APP) containing the dominant NL-G-F mutations with human apoE targeted replacement (TR) mice and used middle-aged NL-G-F/E3 and NL-G-F/E4 mice to assess the role of prior WNV (subtype Kunjin virus) (KUNV) exposure on hAPP/Aβ-induced behavioral alterations, cognitive injury, circadian body temperatures, viral loads, neuropathology, and transcript levels of four immune measures important in the detrimental effects of KUNV on brain function.

**Results:**

KUNV affected physiological, behavioral, cognitive, amyloid pathology, viral load, and immune measures in middle aged NL-G-F mice in an apoE isoform-dependent fashion. NL-G-F/E4 mice were more susceptible to KUNV induced cognitive injury and prolonged viral load in the cortex.

**Discussion:**

These results support an important apoE isoform-dependent role in modulating phenotypes in the NL-G-F AD mouse model following WNV exposure.

## 1 Introduction

Several hypothesis have been proposed about the potential mechanisms underlying of how Alzheimer’s disease (AD) progresses and how to treat it (Powell, 2019). These hypotheses include the viral hypothesis of AD, in particular Herpes Simplex Virus-1, cytomegalovirus, Ljungan virus, and Human Immunodeficiency Virus (Naughton et al., 2020). In addition, West Nile Virus (WNV) infection can cause severe and long-lasting neurological disease (Fulton et al., 2020; Kumar et al., 2016; Samaan et al., 2016; Byas and Ebel, 2020), and infection in the hippocampus results in some of the neuropathology and neuroinflammation seen in AD (Fulton et al., 2020). For example, neuropathology in brains of AD and WNV patients is associated with T cell infiltration in to the brain, activated microglia, production of inflammatory cytokines, and increased permeability of the blood–brain barrier (Gate et al., 2020; Unger et al., 2020; Sweeney et al., 2018; Merlini et al., 2018). In a murine model of WNV-induced neurological sequalae, spatial learning deficits are associated with interferon γ expression from infiltrating CD8 + T cells (Vasek et al., 2016; Garber et al., 2019). Exposure to WNV may therefore impact AD-relevant behavioral and cognitive performance and neuropathology via AD-susceptibility genes and by inducing neuroinflammation in the hippocampus.

There are three human apolipoprotein E (E) isoforms, which play a role in cholesterol metabolism: E2, E3, and E4. Compared to E3, E2 is protective with regard to AD risk (Raber et al., 2004; Spinney, 2014), more prevalent among centenarians, and associated with improved episodic memory performance, larger hippocampal volume, and reduced hippocampal atrophy (Chiang et al., 2010; Goldberg et al., 2020). Episodic memory is associated with the hippocampus and entorhinal cortex that play a key role in AD pathology (Braak and Braak, 1991; Braak and Braak, 1998; Thal et al., 1999). Compared to E3, E4 is an AD risk factor and associated with accelerated hippocampal atrophy (Li et al., 2016). ApoE binds to triggering receptor expressed on myeloid cells 2 (TREM2; Wolfe et al., 2018). TREM2 in microglia (Ulland et al., 2017; Lee et al., 2018) is proposed to be involved in AD-related neuropathology (Prokop et al., 2019; Bonstanciklioglu, 2019). E4 modifies the associations of the angiotensin-converting enzyme (ACE) polymorphisms with neuropsychiatric syndromes in AD (de Oliviera et al., 2017). ACE2 is the entry receptor of SARS-CoV-2 (Oudit et al., 2023), *APOE* genotype was associated with survival in patients infected with COVID19 (Ostendorf et al., 2022) and E4 was associated with severe COVID19 with more prevalent microhemorrhages in intensive care patients (Kurki et al., 2021). Evidence suggest that these apoE isoform differences involve very low-density lipoproteins (VLDL), as E4 binds better than E3 to VLDL and impairs their lipolytic processing (Phillips, 2014). WNV capsid protein binds VLDL (Martins et al., 2019), as does dengue virus capsid protein (Faustino et al., 2014) and is thought to be important for uptake and transport of virus. These data indicate that there are overlapping mechanisms underlying detrimental effects of some distinct neurotropic viruses involving apoE receptors. However, different mechanisms might be involved in the detrimental effects of other neurotropic viruses that show a role of E in the pathogenesis. E4 is also associated with enhanced entry of human immunodeficiency virus 1 (HIV-1) cell entry and HIV-1 disease progression (Burt et al., 2008). E is an HIV-1-inducible inhibitor of viral production and infectivity in macrophages (Siddiqui et al., 2018). E is also involved in the pathogenesis and susceptibility to other infectious diseases, including herpes simplex virus-1, hepatitis C virus, hepatitis E virus, varicella zoster virus, Epstein–Barr virus, malaria, *Listeria monocytogenes* (LM), and *Klebsiella pneumoniae* (Siddiqui et al., 2018). In a mouse model of herpes simplex virus 1 (HSV-1), the cerebral load of latent HSV-1 genomic copies, which is associated with the reactivation risk (Hoshino et al., 2007), is 10-fold higher in E4 than E3 mice (Burgos et al., 2006).

The differential interactions of apoE isoforms with human amyloid precursor protein (APP) and with the amyloid peptides Aβ40 and Aβ42 generated from APP have been proposed to influence cognitive injury (Raber et al., 2000) and neurodegeneration (Buttini et al., 2002). Evidence supports that APP, Aβ40 and Aβ42 are important in viral infections as well. APP binds the HIV-1 gag protein, retains it in lipid rafts and blocks HIV-1 virion production and spread (Chai et al., 2017). The HIV-1 gag protein induces the generation of Aβ40 and Aβ42 and amyloid is elevated in HIV-1 infected brains and binds HIV-1. Aβ42 inhibits influenza A viral replication (White et al., 2014) and might play a role in WNV replication as well. Thus, AD patients might have an altered susceptibility to viral infections, which are hypothesized as triggers and risk factors for developing AD (Rippee-Brooks et al., 2024).

We recently reported that six-month-old (hAPP) mice, which contain the Swedish, Iberian, and Arctic mutation (APP NL-G-F) (*App^NL-G-F^*), show impaired cognitive performance that is associated with increased hippocampal DNA methylation of a 1 Kb region overlapping the 3’UTR of the *Tomm40* gene and the promoter region of the *Apoe* gene (Kundu et al., 2021), both genes that modulate AD risk (Roses et al., 2016).

We crossed knock-in (KI) mice expressing human amyloid precursor protein (APP) containing the dominant NL-G-F mutations (Saito et al., 2016; Saito et al., 2014) with human apoE targeted replacement (TR) mice (Sullivan et al., 1997; Knouff et al., 1999). We used middle-aged NL-G-F/E3 and NL-G-F/E4 mice (Holden et al., 2022) to assess the role of prior WNV exposure on hAPP/Aβ-induced behavioral alterations, cognitive injury, circadian body temperatures, and neuropathology and whether these effects are apoE isoform-dependent and associated with enhanced neuroinflammation in the hippocampus or cortex. We also analyzed viral loads in the cortex and hippocampus 7 weeks after inoculation. The cytokine interferon-γ (IFN-γ) is required to clear RNA viruses like WNV from the brain (Klein et al., 2019). The cytokine tumor necrosis factor-α (TNF-α) might play a pathogenic role in initiating inflammation following WNV infection and contribute to prolonged inflammation following WNV infection (Leis et al., 2020). The chemokine C-X-C motif chemokine ligand 10 (CXCL10 or IFN-γ induced protein 10) is secreted by neurons following WNV infection and help recruiting CD8 + T cells to the brain which in turn help clearing the virus from infected neurons (Klein et al., 2005). We measured T-cell receptor-α (TCR-α) as a surrogate for T cell infiltration into the brain post viral infection (Kitaura et al., 2011). Therefore, we also analyzed the transcript levels of these four immune measures (i.e., TCR-α, IFN-γ, CXCL10, and TNF-α) in the cortex 7 weeks after inoculation.

## 2 Materials and methods

### 2.1 Mice and implantation of temperature sensors

Targeted Replacement (TR) E3 (Sullivan et al., 1997) and E4 (Knouff et al., 1999) mice crossed with NL-G-F mice (Saito et al., 2014) and backcrossed to only contain human APP and human apoE (Holden et al., 2022; Kundu et al., 2022) were used for the current study (*n* = 44 mice; *n* = 15 NL-G-F/E3 mice (*n* = 7 PBS; *n* = 8 KUNV; *n* = 5 males; *n* = 10 females) and *n* = 29 NL-G-F/E4 mice (*n* = 14 PBS; *n* = 15 KUNV; *n* = 4 males; *n* = 25 females). Mice were implanted with temperature sensors. TS100 millimeter-scale (7.5 × 7.5 × 4.2 mm) CubiSensTM wireless sensors (CubeWorks, Ann Arbor, MI), packaged in bio-compatible epoxy and coated with parylene, were implanted under the skin for accurate, real-time temperature measurement. The TS100 is capable of transmitting up to 100 m in distance, last up to 2 years in sensing operation, and allows measuring circadian body temperature in group-housed mice, including during the 4-week period following inoculation we are not allowed access to the mice in the ABSL-2 facility. The sensors were sterilized using the Cidex solution (CubeWorks, Ann Arbor, MI). A heating pad and bead sterilizer were used for the surgeries. For the surgery, the mice were anesthetized with Isoflurane (4% for induction of the anesthesia and 1–3% for maintenance of the anesthesia). Lidocaine (6 mg/kg of 0.5%) was injected subcutaneously around the incision site, immediately prior to the aseptic preparation of the abdomen. To close the skim, 9 mm AUTOCLIP stainless steel clips were used. For pain control, Meloxicam (10 mg/kg) orally prior to the induction of anesthesia and every 24 h for two additional days was used. The mice were treated as indicated below. Body temperatures were acquired and analyzed for prior and during the behavioral testing. Due to technical issues, in NL-G-F/E3 mice no data were saved for days 19–22 and 29–30, and in NL-G-F/E4 mice on day 22. Homozygous breeding of the mice was used to generate the experimental mice for this study. Throughout testing, all the mice were group-housed with mice in the same cage receiving the same treatment. Animals were maintained on a 1,200 h light/dark schedule (lights on at 06:00). Laboratory chow (PicoLab Rodent diet 20, # 5053; PMI Nutrition International, St. Louis, MO, USA) and water were provided *ad libitum*. Behavioral testing took place during the light cycle. All procedures complied with the National Institutes of Health Guide for the Care and Use of Laboratory Animals and with IACUC approval at Oregon Health & Sciences University. Experimenters were blinded to the genotype, sex, and treatment of the mice.

### 2.2 Virus and infections

WNV_KUNV,_ abbreviated KUNV hereaffter) was obtained from BEI Resources, (NIAID, NIH: Kunjin Virus, MRM 16, NR-51653). Viral stocks were grown on C6/36 cells, followed by centrifugation of culture supernatant through a 20% sorbitol cushion at 30,000 rpm for 1.5 h in an SW32 rotor (Beckman). Following centrifugation, virus was resuspended in 1/100 of the original volume in DMEM, and aliquots were stored at −80°C. Viral titers were determined by focus-forming assay on Vero cells. Mice were infected intra-peritoneally (i.p.) with 1,000 focus-forming units (ffu) KUNV (*n* = 23 mice) or PBS control (*n* = 21 mice). We have determined that this dose is sub-lethal in most infected mice. The mice were 13.79 ± 0.53 (PBS) and 13.39 ± 0.51 (KUNV) months of age and there was no different in age in the treatment groups in either genotype. The mice underwent behavioral testing, starting 30 days post infection (dpi), as described below.

### 2.3 Behavioral testing

**Figure 1:**
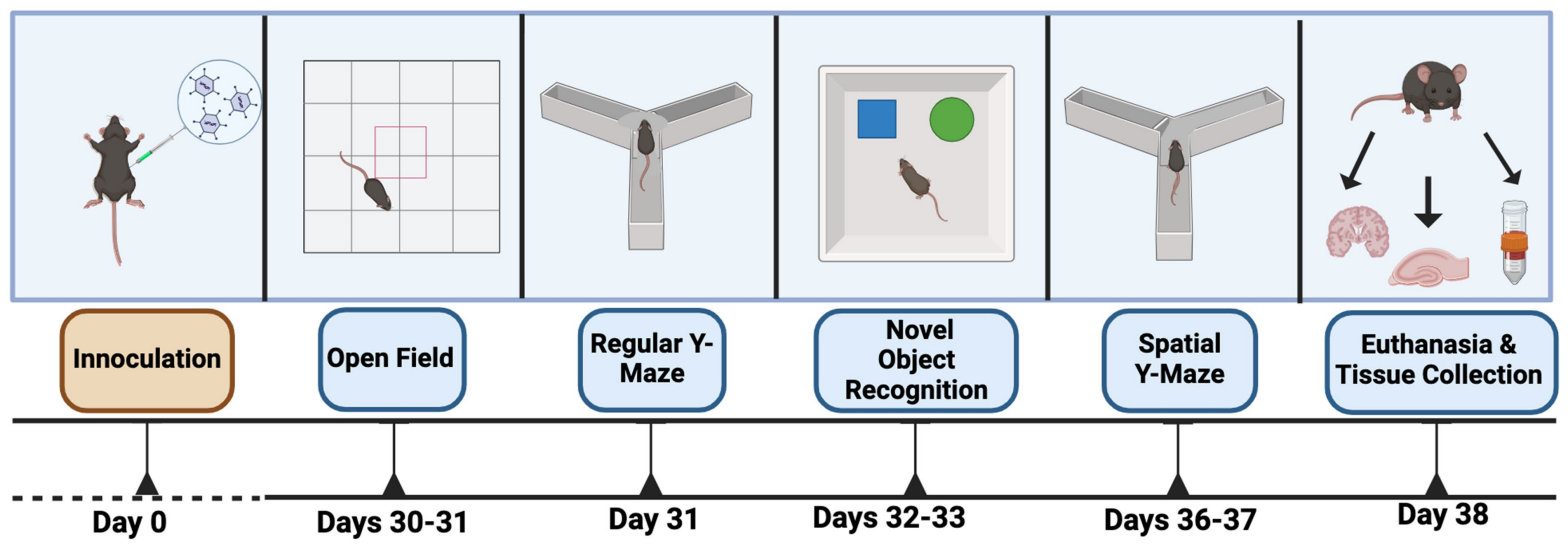
Schedule of behavioral testing. For details, see text.

Mice were behaviorally tested as illustrated in Figure 1. In the mornings of days 30 and 31, mice were tested for measures of activity, measures of anxiety, and spatial habituation in the open field. In the afternoon of day 31, mice were tested for spontaneous alternation in the Y maze. On days 32 and 33, the mice were tested for object recognition. On days 36 and 37, the mice were tested in the spatial Y maze. On day 38, the mice were euthanized by cervical dislocation and blood was collected in EDTA-containing tubes and the hippocampus and cortex from each hemibrain were dissected.

### 2.4 Open field and novel object recognition

**Figure 2:**
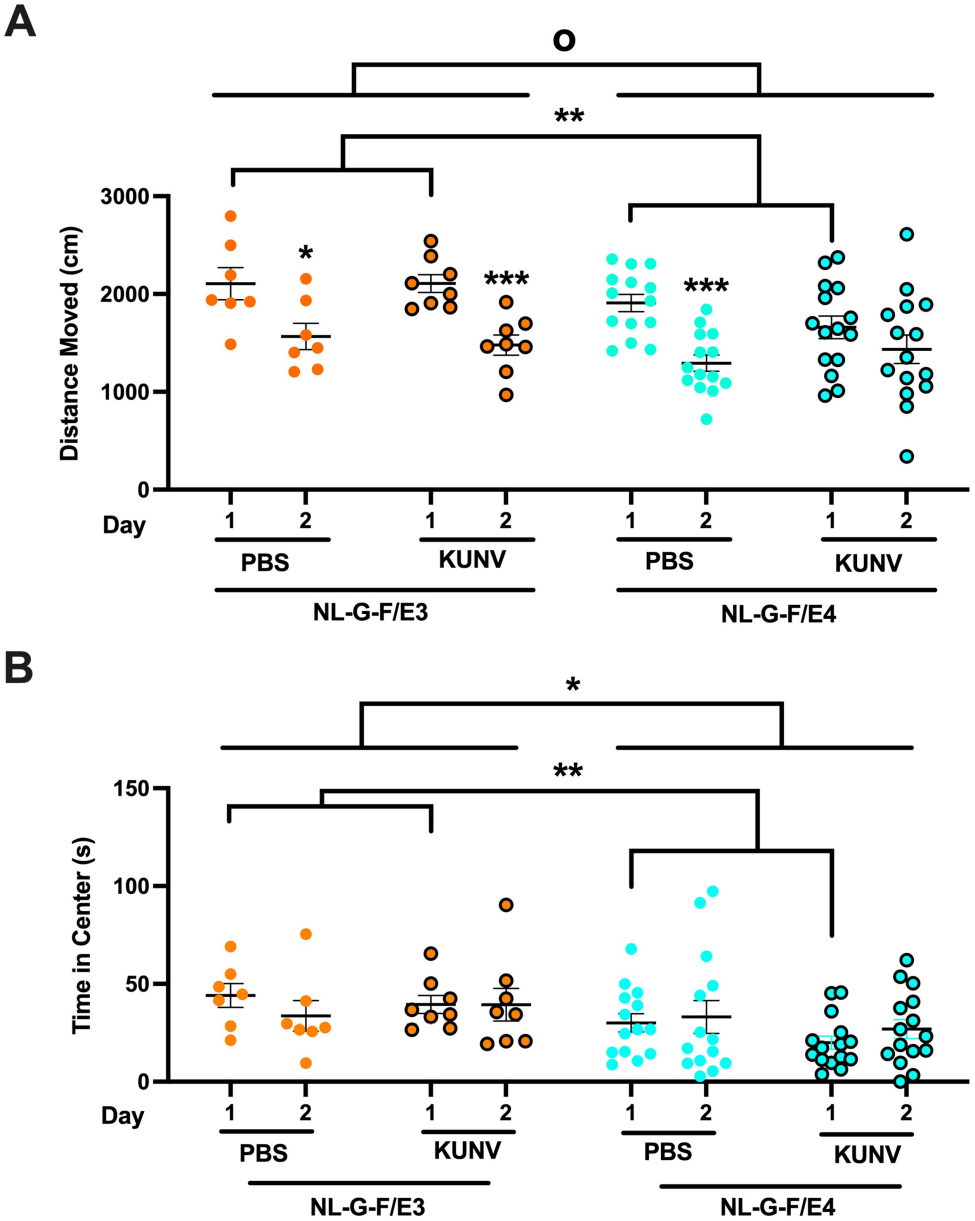
**(A)** Activity levels of PBS- and KUNV-infected NL-G-F/E3 and NL-G-F/E4 mice in the open field. **(B)** Anxiety levels of PBS- and KUNV-infected NL-G-F/E3 and NL-G-F/E4 mice in the open field. PBS-treated E3 mice (*t* = 3.278, *p = 0.0169, paired t-test), KUNV-infected E3 mice (*t* = 5.535, ***p = 0.0009, paired t-test), and PBS-treated NL-G-F/E4 mice (*t* = 5.473, ***p = 0.0001, paired t-test) showed spatial habituation learning but KUNV-infected NL-G-F/E4 mice did not. There was also an effect of genotype [*F*(1,39) = 5.809, ^o^*p* = 0.021], with lower activity levels in NL-G-F/E4 than NL-G-F/E3 mice. For activity levels on day 1 in the open field, there was an effect of genotype [*F*(1,44) = 9.102, **p = 0.004], with higher activity levels in NL-G-F/E3 than NL-G-F/E4 mice. NL-G-F/E3. PBS: *n* = 7; KUNV: *n* = 8; NL-G-F/E4. PBS: *n* = 14; KUNV: *n* = 15.

**Figure 3:**
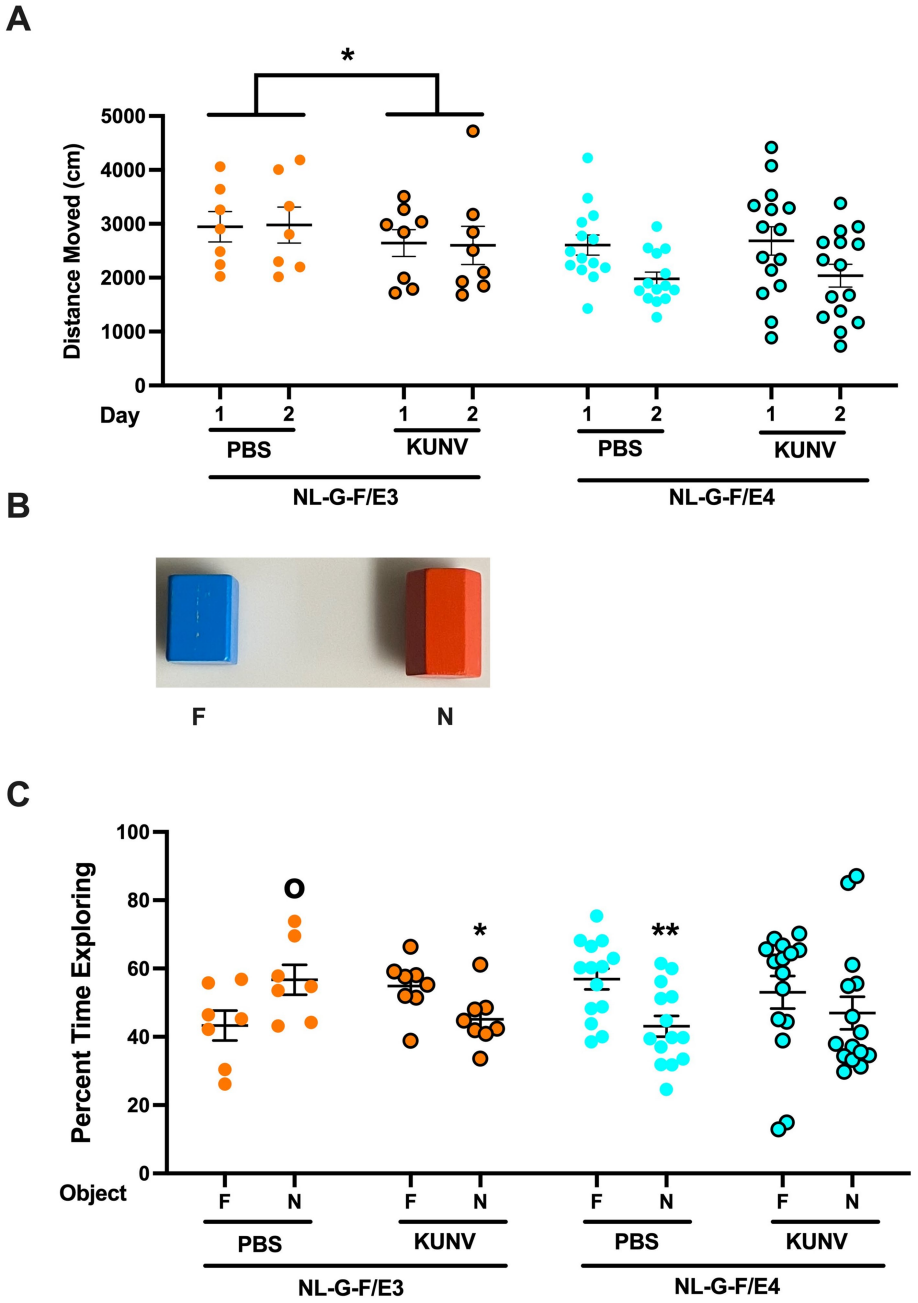
**(A)** Activity in the open field in the presence of the two objects. In NL-G-F/E3 mice, there was an effect of treatment [*F*(1,12) = 5.472, *p = 0.037], with lower activity in KUNV-infected than PBS-treated NL-G-F/E3 mice. In NL-G-F/E4 mice, there were no significant effects. **(B)** During the training trial, the mice were exposed to two identical objects; during the test trial one of the familiar objects (F) was replaced by a novel one (N). **(C** Object recognition of PBS- and KUNV-infected NL-G-F/E3 and NL-G-F/E4 mice. PBS-treated NL-G-F/E3 mice (*t* = 2.153, ^o^*p* = 0.0262) showed novel object recognition and spent significantly more time exploring the novel than familiar object. KUNV-infected NL-G-F/E3 mice and PBS-treated NL-G-F/E4 mice (*t* = 2.431, *p = 0.0291) spent significantly more time exploring the familiar than novel object, while KUNV-infected NL-G-F/E4 mice did not show a preference for exploring either the novel or familiar object. PBS: *n* = 7; KUNV: *n* = 8; NL-G-F/E4. PBS: *n* = 14; KUNV: *n* = 15.

The mice were put in an open field enclosure (16 × 16 inches, Kinder Scientific, Poway, CA) for 10 min on two subsequent days (Figure 2). On day 3, the open field contained two identical objects for a 15-min trial. The objects were placed 10 cm from each other. The next day, one object was replaced with a novel object for a 15-min trial. Images of the actual objects used are illustrated in Figure 3B. Between trial, the arenas and objects were cleaned with 0.5% acetic. Interaction with the object was coded as object exploration (i.e., nose sniffing the objects) by hand scoring videos acquired with Noldus Ethovision software (version 17, Wageningen, The Netherlands). The percent time spent exploring the familiar object and the novel object was analyzed. Different objects were used during the first and second week of testing.

The outcome measures in the open field analyzed were: (1) distance moved in the open field in the absence and presence of objects, an activity measure; (2) the difference in the distance moved in the open field over days, habituation to the open field, a cognitive measure; (3) time spent in the center of the open field, an anxiety measure; and (4) percent time spent exploring the familiar and novel object in the object recognition test, a cognitive measure.

### 2.5 Regular Y maze

Activity levels and hippocampus-dependent spontaneous alternations were assessed in the Y-shaped maze from O′ Hara & Co., Ltd. (Tokyo, Japan) had raised sides (3.8 cm bottom width, 12.55 cm top width, 12.55 cm height) with plastic, opaque gray arms (37.98 cm length). the Y-maze (O′ Hara & Co., Ltd., Tokyo, Japan) in a 5-min trial. The maze was cleaned with 0.5% acetic acid between trials. Performance was recorded using Noldus Ethovision software and hand scoring used to assess the number of arm entries and the percent spontaneous alternations. The outcome measures in the Y maze were total arm entries, an activity measure, and percent spontaneous alternations, a cognitive measure.

### 2.6 Spatial Y maze

The spatial Y-maze test was conducted using smaller and distinct Y-mazes from those used for assessment of spontaneous alternation (Harvard Apparatus, Panlab, Holliston, MA, United States). This task was conducted over 2 consecutive days. On day 1, one arm was blocked off and mice were allowed to explore the maze for 15 min. Extra-maze spatial cues were taped on all three walls of the biosafety cabinet in which the mice were being tested. On day 2, all arms were accessible, and mice were allowed to explore for a 5-min trial. Performance of the mice was tracked using Ethovision software (version 17). Digital videos of day 2 were later analyzed to measure the number of entries into and the percent time spent in the novel arm (the arm that was blocked off during day 1) out of the entries in all three arms in trial 2. The criterion for an arm entry was when all four limbs were within the arm.

### 2.7 Gene expression, viral load and ab analyses

Following euthanasia by quick cervical dislocation without anesthesia, trunk blood was collected in EDTA-coated tubes, and brains were dissected and RNA isolated from cortical and hippocampal tissue of one hemisphere. RNA isolated from hippocampi and cortices was analyzed by qRT-PCR for expression of the inflammatory mediators TNF-α (assay ID: Mm00443258_m1), IFN-γ (Μm01168134_m1), CXCL10 (Mm00445235_m1) and T cell receptor α (Mm01313019_g1) using predesigned primer/ probe sets (Thermofisher). RNA was extracted using TRIzol reagent (Invitrogen) according to manufacturer’s protocol. Relative expression of cytokines was determined by qRT-PCR using gene specific primer-probe sets (ThermoFisher) and normalized to β-actin mRNA expression (Mm00607939) using the ΔΔCt method (Livak and Schmittgen, 2001). Viral genomes were quantified using WNV_KUNV_ specific primer probes. The primer/probe sequences for KUNV detection and quantitation are:

Forward Primer 5’-AGTGGAGAAGTGGAGCGATGTT-3’

Reverse Primer 5’-CAGGCTGCCACACCAAATG-3’

Probe FAM-CATACTCTGGCAAACGA-MGB

Relative expression levels (RQ) were calculated by the ∆∆C_T_ method as previously described (Livak and Schmittgen, 2001). Briefly, Ct values were obtained in duplicate for the gene of interest (GOI) and β-actin in each sample. ∆C_T_ (Avg. GOI C_T_ − β-actin C_T_) and ∆∆C_T_ (∆C_T, Sample_ − ∆C_T, reference control sample_) were calculated for each sample. Relative quantitation = 2^-∆∆C^_T._

The cortex of the other hemisphere was processed for analyses of soluble and insoluble Aβ. Cortices were processed for analyses of soluble and insoluble Aβ40 and Aβ42 levels using MyBiosource ELISA kits (catalog numbers MBS760432 and MBS268504, respectively; San Diego, MA, United States), according to the recommended guidelines in the production information sheets. To a thawed cortical tissue sample (both hemispheres), 200 μl of buffer A (phosphate-buffered saline containing a protease inhibitor tablet (cOmpleteTM, 11,836,170,001 Roche, Millipore Sigma, Burlington, MA, United States and filtered before use) was added. The tissue was homogenized using a Polytron for 10 s and subsequent a sonicator and centrifuged at 45,000 rpm for 20 min at 4°C. The supernatant was collected as the soluble fraction. The same volume of buffer A was used to loosen the pellet. The sample was centrifuged again at 45,000 rpm for 5 min at 4°C. After removing the supernatant in a separate tube. The pellet was dissolved in Buffer B (containing 6 M Guanidine H-Cl and 50 mM Tris and filtered before use) and incubated at room temperature for 1 h. After this incubation, the sample was sonicated for 20 s and the extracted pellet was centrifuged at 45,000 rpm for 20 min at 4°C. The supernatant was collected as the insoluble fraction. For each ELISA, pilot experiments were performed to determine the optimal sample dilution to assure that the optical density was within the range of the standard curve. For the analyses of insoluble Aβ levels, the tissue samples were diluted 1: 4,000. For analysis of the insoluble Aβ levels, undiluted tissues samples were used. Standard curves were generated with the same buffer dilution as the samples. The ELISAs were read at 450 nm using a SpectraMax iD5 Multi-Mode Microplate Reader (Molecular Devices, VWR 76175–474, San Jose, CA, United States). The standard curves were generated and the levels in the samples determine using GraphPad Prism software, San Diego, CA, United States). Total protein amounts in the samples were determined by BCA protein assay kit (Pierce, Thermo Scientific, catalog #23225, Waltham, MA, United States) and reading the samples at 562 nm using the iD5 Reader.

### 2.8 Statistical analyses

All behavioral data are reported as mean ± standard error of the mean and were analyzed using SPSS v.22 (IBM, Armonk, NY, USA) or GraphPad v.8 (La Jolla, CA, USA) software. Genotype and treatment were included as factors in analysis of variance (ANOVAs). Sex was used as a covariate in this study, as there were not sufficient mice of each sex to include sex as a factor. In case there were statistical interactions, genotypes were analyzed separately, as indicated. Repeated-measures ANOVAs were used when appropriate. For analysis of object recognition, the percent time spent exploring the familiar and novel object in each group was analyzed. For the circadian data, based on the pattern of the data, the light and dark periods were analyzed as separate analyses, with the mean body temperature in the light or dark period of each day as the repeated measure. Statistical significance was considered as *p* < 0.05. When sphericity was violated (Mauchly’s test), Greenhouse–Geisser corrections were used. Mice were tested in separate cohorts, each containing mice of all experimental groups. All researchers were blinded to genotype and treatment and the code was only broken after the data were analyzed.

## 3 Results

### 3.1 Viral infection

Mice were inoculated with the KUNV subtype of WNV. Although this virus is less lethal than WNV_NY99_ or other recent lineage I isolates from the U. S. or Europe, it is capable of neuroinvasion in C57Bl/6 background mice (Donadieu et al., 2013). Mice were inoculated intraperitoneally with 1,000 ffu KUNV. Subsequent experiments were performed as shown in Figure 1.

### 3.2 Open field and novel object recognition

When the activity levels in the open field were analyzed over the two subsequent days, there was an overall effect of day 1 [*F*(1,39) = 10.583, *p* = 0.002] (Figure 2A). PBS-treated E3 mice (*t* = 3.278, *p* = 0.0169, paired t-test) and KUNV-infected E3 mice (*t* = 5.535, *p* = 0.0009, paired t-test) showed spatial habituation learning and moved less on day 2 than day 1. However, while spatial habituation was significant in PBS-treated E4 mice (*t* = 5.473, *p* = 0.0001, paired t-test), in KUNV-infected E4 mice, it was not (*t* = 1.1617, *p* = 0.1281, paired t-test). There was also an effect of genotype [*F*(1,39) = 5.809, *p* = 0.021], with lower activity levels in NL-G-F/E4 than NL-G-F/E3 mice. There was also a trend towards a day x genotype x treatment interaction [*F*(1,39) = 10.583, *p* = 0.056]. As the open field is a novel environment on day 1, performance on day 1 was also analyzed separately. For activity levels on day 1 in the open field, there was an effect of genotype [*F*(1,44) = 9.102, *p* = 0.004].

When time in the more anxiety-provoking center of the open field was analyzed, there was an effect of genotype [*F*(1,39) = 5.582, *p* = 0.023] (Figure 2B). For time spent in the center of the open field on only day 1, there was also an effect of genotype [*F*(1,44) = 10.819, *p* = 0.002].

The objects used in the object recognition test are illustrated in Figure 3B. When activity in the open field in the presence of the two objects was analyzed, there was a day x genotype interaction [*F*(1,39) = 14.864, *p* < 0.001] (Figure 3A). In NL-G-F/E3 mice, there was an effect of treatment [*F*(1,12) = 5.472, *p* = 0.037], with lower activity in KUNV-infected than PBS-treated NL-G-F/E3 mice. In addition, there was an effect of sex [*F*(1,12) = 6.000, *p* = 0.031], with slightly higher activity levels in female (mean distance moved: 2512 cm) than male (mean distance moved: 2451 cm) mice. In NL-G-F/E4 mice, there were no significant effects.

When time spent in the center of the open field containing objects was analyzed, there was a trend towards a day x genotype interaction [*F*(1,39) = 3.614, *p* = 0.065] (Supplementary Figure S1).

PBS-treated NL-G-F/E3 mice showed novel object recognition and spent significantly more time exploring the novel than familiar object (*t* = 2.153, *p* = 0.0262; Figure 3B). In contrast, KUNV-infected NL-G-F/E3 mice spent significantly more time exploring the familiar than novel object (*t* = 2.431, *p* = 0.0291; Figure 3B). PBS-treated NL-G-F/E4 mice also spent significantly more time exploring the familiar than novel object (*t* = 3.206, *p* = 0.0036; Figure 3B). However, KUNV-infected NL-G-F/E4 mice did not a preference for exploring either the novel or familiar object (Figure 3B).

### 3.3 Regular Y maze

**Figure 4:**
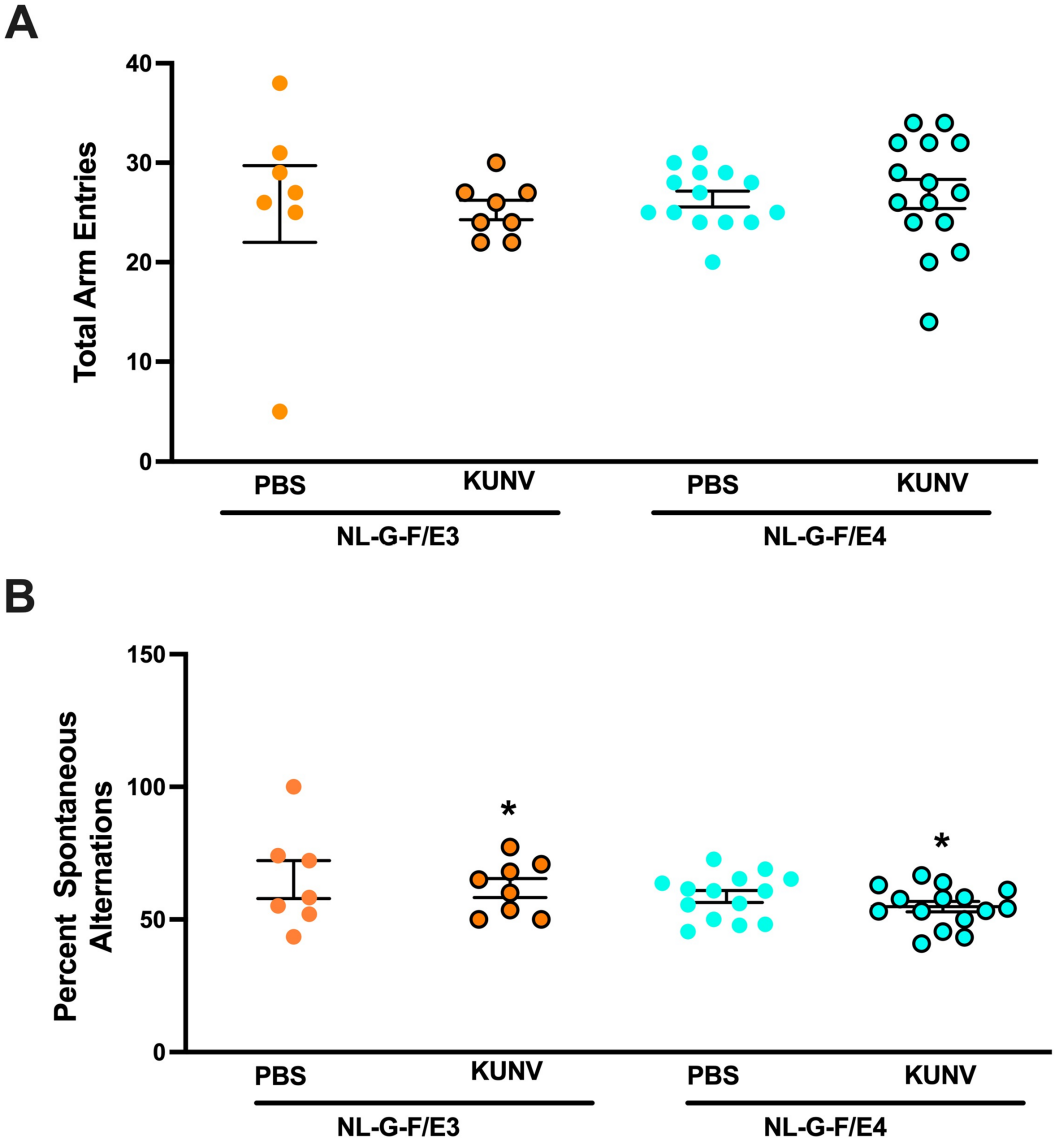
**(A)** Activity levels in the Y maze. All groups showed comparable activity levels in the Y maze. **(B)** Spontaneous alternation in the Y maze. There was an effect of treatment [*F*(1,44) = 4.785, *p = 0.035], with less spontaneous alternation in KUNV- than PBS-treated mice. PBS: *n* = 7; KUNV: *n* = 8; NL-G-F/E4. PBS: *n* = 14; KUNV: *n* = 15.

When the number of entries was analyzed as activity measure, there were no significant effects (Figure 4A). However, when spontaneous alternation was assessed in the Y maze, there was an effect of treatment [*F*(1,44) = 4.785, *p* = 0.035], with less spontaneous alternation in KUNV infected—than PBS-treated mice (Figure 4B). There was also an effect of sex [*F*(1,44) = 5.487, *p* = 0.024], with slightly higher spontaneous alternation in females (61%) than males (57%).

### 3.4 Spatial Y maze

**Figure 5:**
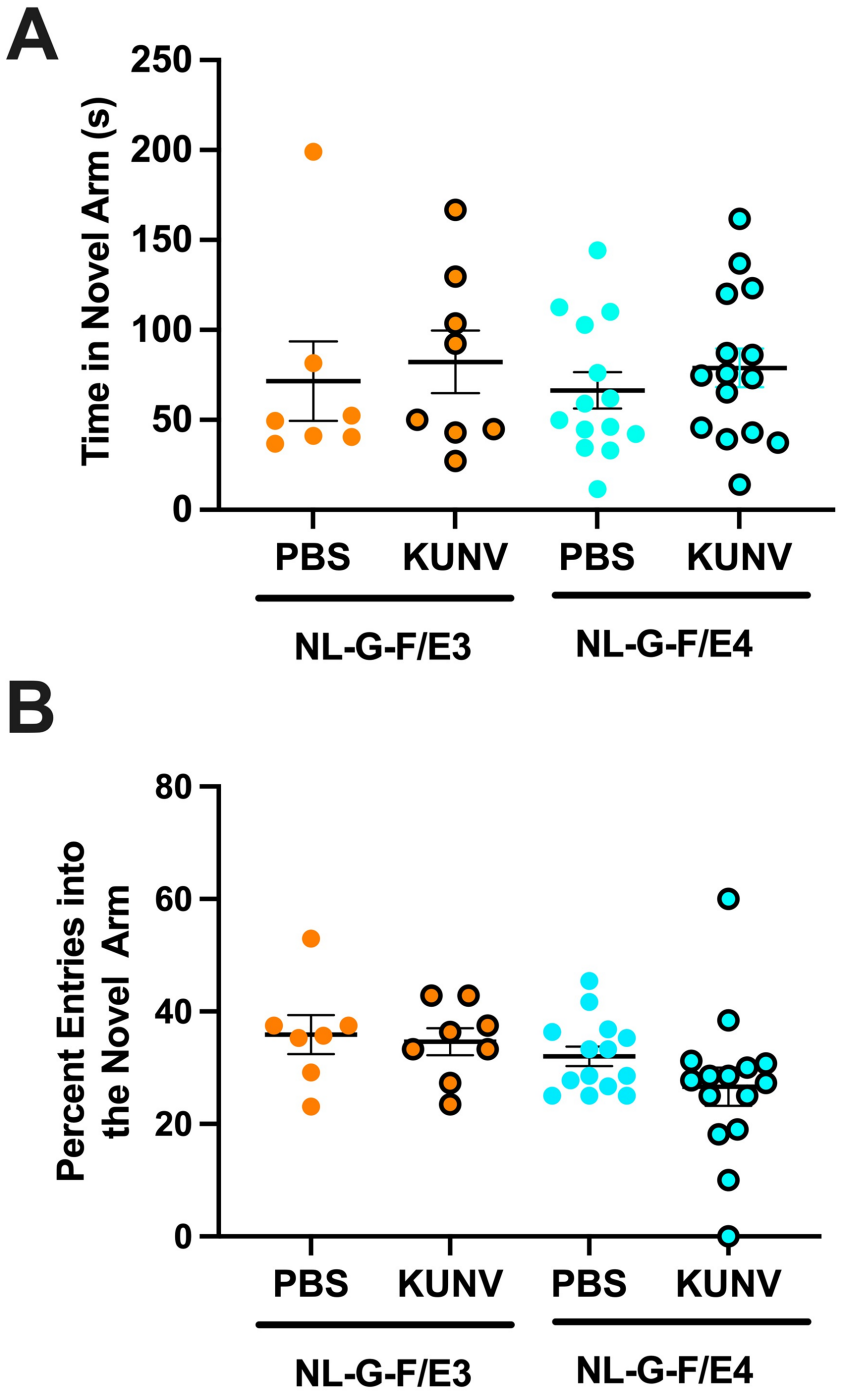
(**A)** Time spent in the novel arm in the spatial 24-h Y maze. All groups spent a comparable time in the novel arm. **(B).** Percent entries in the spatial Y maze. All groups showed a similar percentage of entries into the novel arm. PBS: *n* = 7; KUNV: *n* = 8; NL-G-F/E4. PBS: *n* = 14; KUNV: *n* = 15.

When the time spent in the novel arm of the spatial Y maze was analyzed, there were no significant effects (Figure 5A). When the percent entries in the novel arm when analyzed, there were also no significant effects (Figure 5B).

### 3.5 Circadian body temperatures

**Figure 6:**
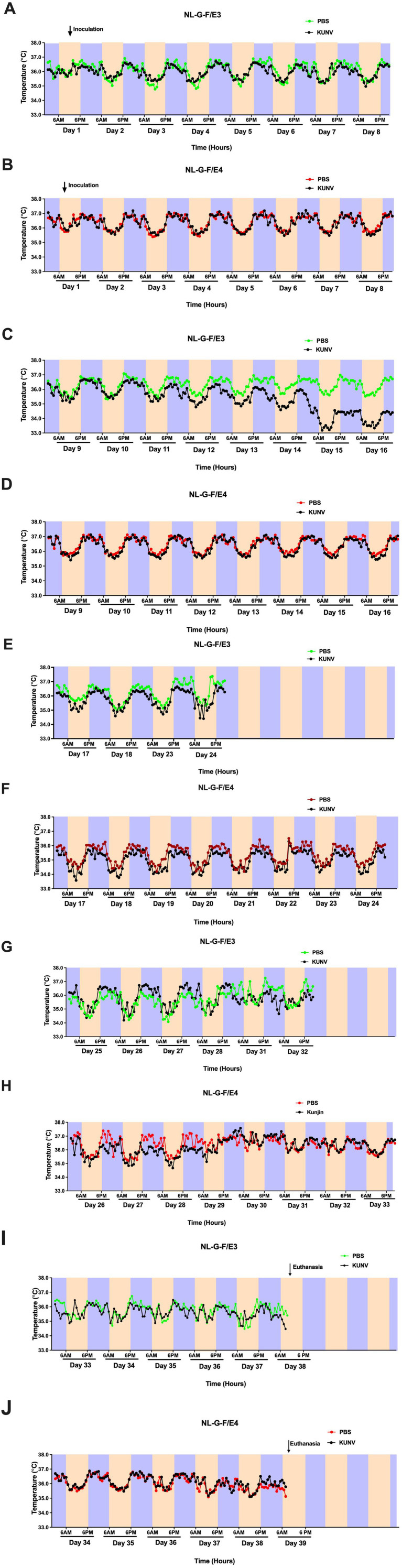
Circadian body temperatures in KUNV- and PBS-treated NL-G-F/E3 **(A, C, E, G)** and NL-G-F/E4 **(B, D, F, H)** mice. Body temperatures 4.5 weeks following PBS- or KUNV-exposure are illustrated. In general, the body temperature was higher in NL-G-F/E4 than NL-G-F/E3 mice and the effect of KUNV on body temperature was more profound in NL-G-F/E3 than NL-G-F/E4 mice, with higher body temperatures in PBS- than KUNV-infected NL-G-F/E3 mice. This effect was most pronounced the week following inoculation, D11-D16, with the effect becoming more pronounced over subsequent days. NL-G-F/E3. PBS: *n* = 6; KUNV: *n* = 7; NL-G-F/E4. PBS: *n* = 11; KUNV: *n* = 14.

The circadian body temperatures are illustrated in Figures 6A–H. The light and dark periods for each were analyzed as separate analyses, with the mean body temperature in the light or dark period of each day as the repeated measure. For each panel, we first analyzed all groups together. Based on the genotype effects and interactions with genotype, we followed up with analyses of each genotype. The detailed statistical analyses are described in the Supplementary data. The body temperature was higher in NL-G-F/E4 than NL-G-F/E3 mice. The effect of KUNV on body temperature was more profound in NL-G-F/E3 than NL-G-F/E4 mice, with higher body temperatures in PBS- than KUNV-infected NL-G-F/E3 mice. This effect was most pronounced the week following inoculation, D11-D16, with the effect becoming more pronounced over subsequent days.

### 3.6 Insoluble and soluble cortical Aβ40, Aβ42, and the Aβ42/40 ratio

**Figure 7:**
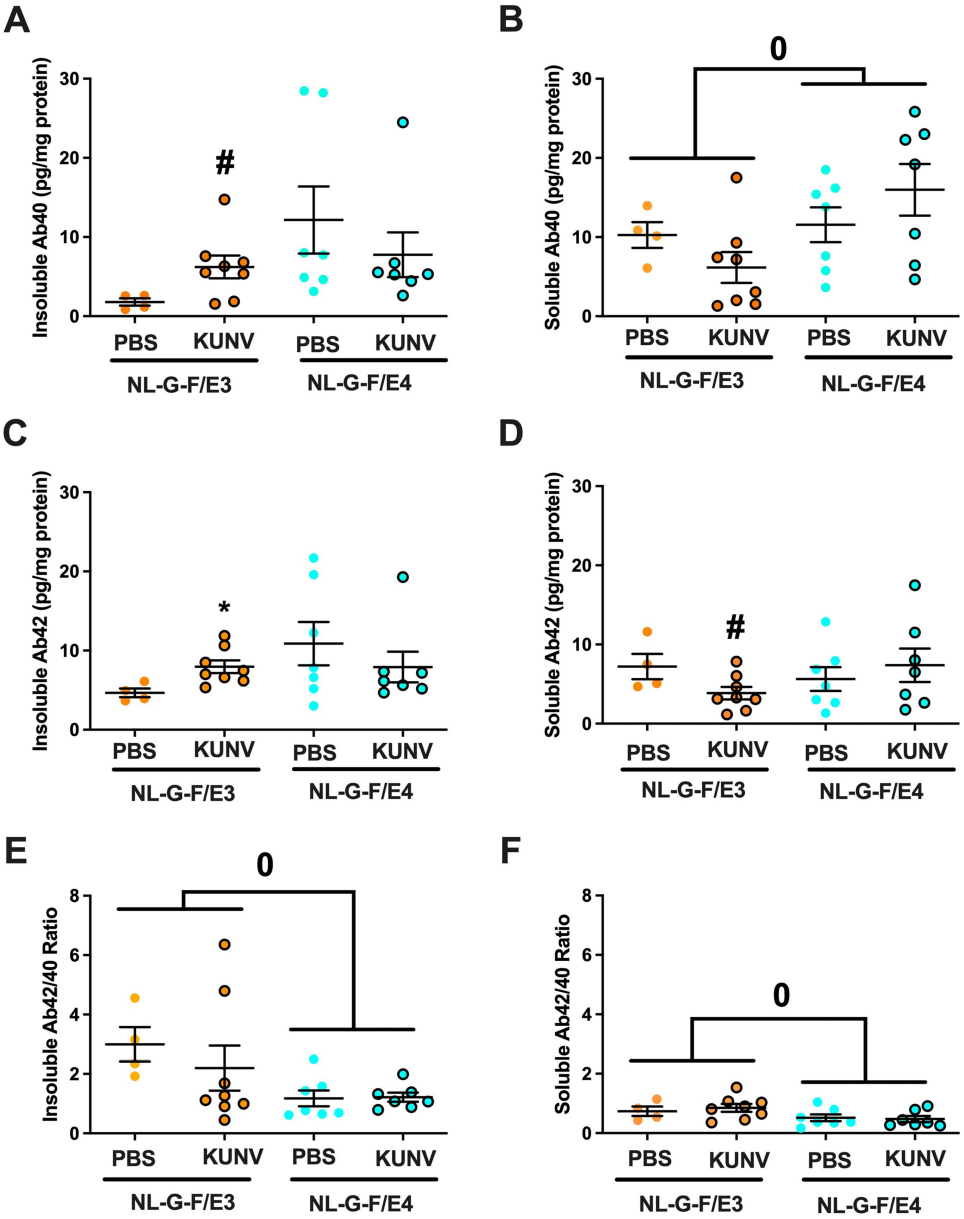
**(A)** There was a trend towards higher insoluble Aβ40 levels in the cortex of KUNV- than PBS-treated NL-G-F/E3 mice (*t* = 2.093, ^#^*p* = 0.0626). This was not seen in NL-G-F/E4 mice. **(B)** There was an effect of genotype on the cortical soluble Aβ40 levels (*F* = 4.609, ^0^*p* = 0.0431), with higher cortical soluble Aβ40 levels in NL-G-F/E4 than NL-G-F/E3 mice. **(C)** Insoluble cortical Aβ42 levels were higher in KUNV- than PBS-treated NL-G-F/E3 mice (*t* = 2.710, ^*^p = 0.0219). This was not seen in NL-G-F/E4 mice. **(D)** There was a trend towards lower soluble Aβ42 levels in KUNV- than PBS-treated NL-G-F/E3 mice (*t* = 2.417, ^#^*p* = 0.0573). This was not seen in NL-G-F/E4 mice. **(E)** There was an effect of genotype on the cortical insoluble Aβ42/40 ratio (*F* = 6.448, ^0^*p* = 0.0187), with a higher cortical insoluble Aβ42/40 ratio in NL-G-F/E4 than NL-G-F/E3 mice. **(F)** There was an effect of genotype on the cortical soluble Aβ42/40 ratio (*F* = 5.140, ^0^*p* = 0.0336), with a higher cortical soluble Aβ42/40 ratio in NL-G-F/E4 than NL-G-F/E3 mice. NL-G-F/E3. PBS: *n* = 4; KUNV: *n* = 8; NL-G-F/E4. PBS: *n* = 7; KUNV: *n* = 7.

Following behavioral testing, insoluble and soluble cortical levels of Aβ40, Aβ42, and the Aβ42/40 ratio were analyzed. In NL-G-F/E3 mice, there was a trend towards higher insoluble Aβ40 levels in the cortex following KUNV than PBS treatment (*t* = 2.093, *p* = 0.0626; Figure 7A). This was not seen in NL-G-F/E4 mice. For cortical soluble Aβ40 levels, there was an effect of genotype (*F* = 4.609, *p* = 0.0431), with higher cortical soluble Aβ40 levels in NL-G-F/E4 than NL-G-F/E3 mice (Figure 7B).

In NL-G-F/E3 mice, insoluble cortical Aβ42 levels were also higher following KUNV than PBS treatment (*t* = 2.710, *p* = 0.0219; Figure 7C). This was not seen in NL-G-F/E4 mice. For cortical soluble Aβ42 levels in NL-G-F/E3 mice, there was a trend towards lower levels following KUNV than PBS treatment (*t* = 2.417, *p* = 0.0573). This was not seen in NL-G-F/E4 mice.

For the cortical insoluble Aβ40/40 ratio, there was an effect of genotype (*F* = 6.448, *p* = 0.0187), with a lower cortical insoluble Aβ42/40 ratio in NL-G-F/E4 than NL-G-F/E3 mice (Figure 7E). For the cortical soluble Aβ42/40 ratio, there also was an effect of genotype (*F* = 5.140, *p* = 0.0336), with a lower cortical soluble Aβ42/40 ratio in NL-G-F/E4 than NL-G-F/E3 mice (Figure 7F).

### 3.7 Viral load in the cortex and hippocampus of NL-G-F/E3 and NL-G-F/E4 mice

**Figure 8:**
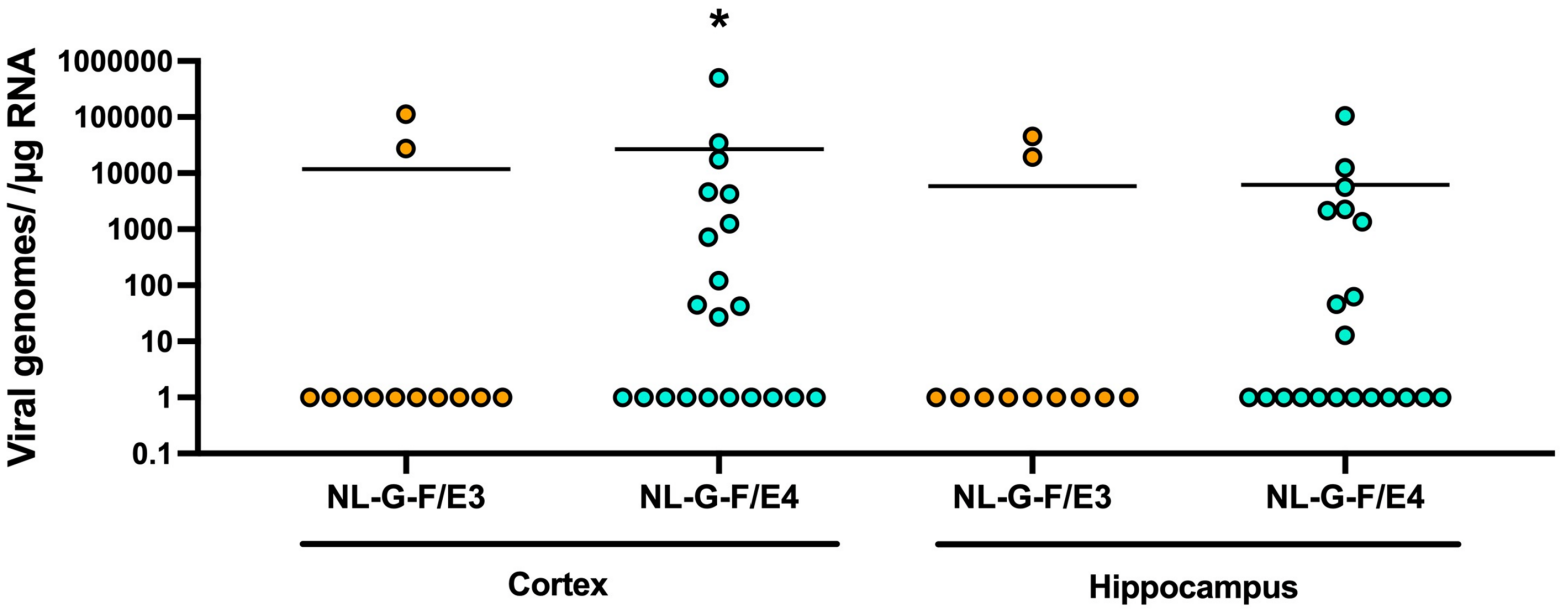
Viral loads in the cortex (left) and hippocampus (right) 7 weeks after KUNV inoculation. KUNV viral load was detected in the cortex of relatively more NL-G-F/E4 than NL-G-F/E3 mice. *p = 0.034, 2-sided Chi-square test. In the hippocampus, this genotype difference did not reach statistical significance. *p* = 0.1628, 2-sided Chi-square test. Cortex. NL-G-F/E3: *n* = 12; NL-G-F/E4: *n* = 21; Hippocampus. NL-G-F/E3: *n* = 11; NL-G-F/E4: *n* = 21.

Next, we assessed viral loads in the cortex and hippocampus 7 weeks after KUNV inoculation. In the cortex, viral load was detected in more NL-G-F/E4 than NL-G-F/E3 mice (Figure 8). In the cortex of KUNV-infected NL-G-F/E3 mice, viral load was detected in only 2 out of the 12 mice, while in the cortex of KUNV-infected NL-G-F/E4 mice, viral load was detected in 11 out of the 21 mice (*p* = 0.034, 2-sided Chi-square test). In the hippocampus of KUNV-infected NL-G-F/E3 mice, viral load was detected in 2 out of the 11 mice, while in the hippocampus of KUNV-infected NL-G-F/E4 mice, viral load was detected in 9 out of the 21 mice but this genotype difference did not reach statistical significance.

### 3.8 Immune measures in the cortex of NL-G-F/E3 and NL-G-F/E4 mice

**Figure 9:**
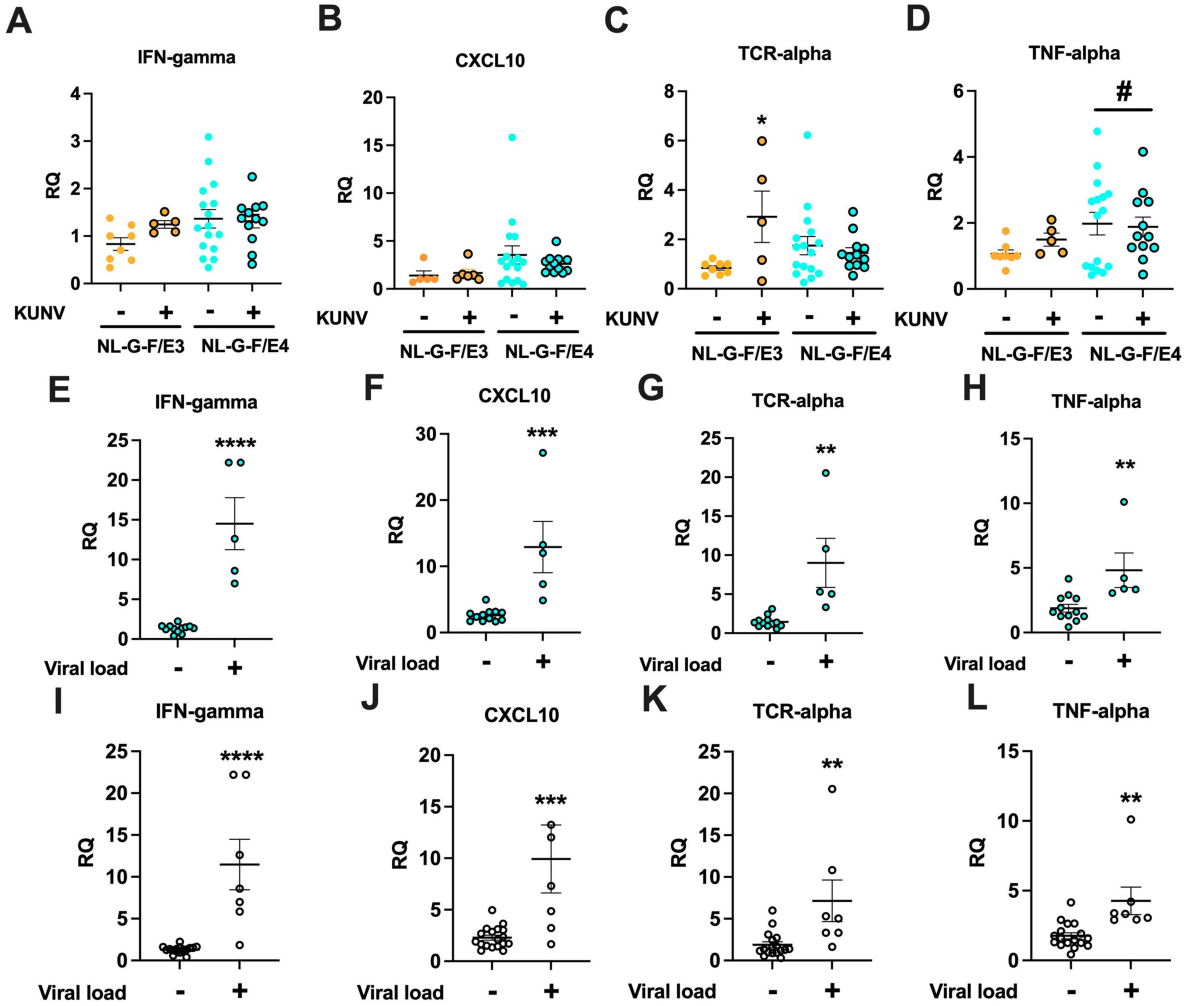
Relative expression levels (RQ) were calculated by the ∆∆C_T_ method as previously described (Livak and Schmittgen, 2001). Ct values were obtained in duplicate for the gene of interest (GOI) and β-actin in each sample. ∆C_T_ (Avg. GOI C_T_ - β-actin C_T_) and ∆∆C_T_ (∆C_T, Sample_- ∆C_T, reference control sample_) were calculated for each sample. Cortical transcripts of IFN-*γ* **(A)**, CXCL10 **(B)**, TCR-*α* **(C)**, and TNF-α **(D)** in PBS-treated NL-F-G/E3 and NL-G-F/E4 mice and KUNV-infected NL-F-G/E3 and NL-G-F/E4 mice that did not show detectable viral loads at 7 weeks post inoculation. For TCR-α there was a genotype x treatment interaction [*F*(1,37) = 7.304, *p* = 0.0103]. TCR-α transcript levels were higher in KUNV-infected NL-F-G/E3 mice that did not show detectable viral loads than PBS-treated NL-G-F/E3 mice (*t* = 2.573, *p = 0.0259). For TNF-α levels, there was a trend towards a genotype difference p*F*(1,37) = 3.207, ^#^*p* = 0.0815], with a trend towards higher TNF-α transcript levels in NL-G-F/E4 than NL-G-F/E3 mice. A. NL-G-F/E3; −: *n* = 8; +: *n* = 5; NL-G-F/E4; −: *n* = 16; +: *n* = 14. B: NL-G-F/E3; −: *n* = 5; +: *n* = 6; NL-G-F/E4; −: *n* = 16; +: *n* = 14. C. NL-G-F/E3; −: *n* = 8; +: *n* = 5; NL-G-F/E4; −: *n* = 16; +: *n* = 14. D. NL-G-F/E3; −: *n* = 8; +: *n* = 5; NL-G-F/E4; −: *n* = 16; +: *n* = 14. Cortical transcript levels IFN-γ **(E)**, CXCL10 **(F)**, TCR-α **(G)**, and TNF-α **(H)** in KUNV-infected NLGF/E4 mice without and with a viral load in the cortex. All four measures were higher in NL-G-F/E4 mice showing a viral load in the cortex than those who did not. **(E)** *t* = 6.531, ****p < 0.0001). **(F)** *t* = 4.267, ***p = 0.0007) **(G)**, *t* = 3.848, **p = 0.0016). **(H)** *t* = 3.102, **p = 0.0073. **(E–H)** −: *n* = 12; +: *n* = 5. Cortical transcript levels IFN-γ **(I),** CXCL10 **(J)**, TCR-α **(K)**, and TNF-α **(L)** in KUNV-infected NL-G-F/E3 and NLGF/E4 mice combined without and with a viral load in the cortex. All four measures were higher in NL-G-F/E3 and NL-G-F/E4 mice combined showing a viral load in the cortex than those who did not. **(I)** *t* = 5.398, ****p < 0.0001. **(J)** *t* = 3.769, ***p = 0.0010. **(K)** *t* = 3.197, **p = 0.0042. **(L)** *t* = 3.564, **p = 0.0017. **(I–L)** −: *n* = 17; +: *n* = 7.

Transcripts of four immune measures were analyzed in the cortex: IFN-*γ*, CXCL10, TCR-*α*, and TNF-α. First, the transcript levels of these four immune measures in cortex were analyzed in PBS-treated NL-F-G/E3 and NL-G-F/E4 mice and KUNV-infected NL-F-G/E3 and NL-G-F/E4 mice that did not show detectable viral loads at seven weeks post inoculation. No significant effects were seen for IFN-γ (Figure 9A) or CXCL10 (Figure 9B). However, for TCR-α there was an effect of treatment [*F*(1,37) = 4.115, *p* = 0.0497] and a genotype x treatment interaction [*F*(1,37) = 7.304, *p* = 0.0103] (Figure 9C). TCR-*α* transcript levels were higher in KUNV-infected NL-F-G/E3 mice that did not show detectable viral loads than PBS-treated NL-G-F/E3 mice (*t* = 2.573, *p* = 0.0259). This pattern was not seen in NL-G-F/E4 mice. For TNF-α levels, there was a trend towards a genotype difference [*F*(1,37) = 3.207, *p* = 0.0815], with a trend towards higher TNF-*α* transcript levels in NL-G-F/E4 than NL-G-F/E3 mice (Figure 9D).

Next, we analyzed whether in KUNV-infected NL-G-F/E4 mice the cortical transcript levels of these measures were higher in mice showing a viral load in the cortex than those who did not. Transcripts of IFN-γ (*t* = 6.531, *p* < 0.0001, Figure 9E), CXCL10 (*t* = 4.267, *p* = 0.0007, Figure 9F), TCR-α (*t* = 3.848, *p* = 0.0016, Figure 9G), and TNF-α (*t* = 3.102, *p* = 0.0073, Figure 9F) were all higher in KUNV-infected NL-G-F/E4 mice showing a viral load in the cortex than those who did not. As only two KUNV-infected NL-G-F/E3 mice showed viral load in the cortex, we also combined NL-G-F/E3 and NL-G-F/E4 mice to assess whether the cortical transcript levels of these measures were higher in mice showing a viral load in the cortex than those who did not. Transcripts of IFN-γ (*t* = 5.398, *p* < 0.0001, Figure 9I), CXCL10 (*t* = 3.769, *p* = 0.0010, Figure 8J), TCR-α (*t* = 3.197, *p* = 0.0042, Figure 9K), and TNF-α (*t* = 3.564, *p* = 0.0017, Figure 9L) were all higher in KUNV-infected mice showing a viral load in the cortex than those who did not.

Relative expression of cytokines was determined by qRT-PCR using gene specific primer-probe sets (ThermoFisher) and normalized to β-actin mRNA expression (Mm00607939) using the ΔΔCt method and expressed as RQ (relative quantification).

## 4 Discussion

The results of KUNV treatment in middle-aged NL-G-F/E3 and NL-G-F/E4 mice are summarized in Table 1. The data of the current study show that exposure to WNV affects physiological, behavioral, cognitive, amyloid pathology, viral load, and immune measures in middle aged NL-G-F mice in an apoE isoform-dependent fashion. KUNV-infected NL-G-F-/E4 mice showed impairments in hippocampus-dependent spatial habituation learning in the open field, increased anxiety levels in the open field, impairments in the ability to distinguish the two objects in the novel object recognition test, more mice with viral loads in the cortex 7 weeks after exposure than seen in the cortex of KUNV-infected NL-G-F/E3 mice. In contrast, KUNV-infected NL-G-F/E3 mice showed lower activity levels in the open field containing objects, neophobia (extreme fear of anything novel) in the object recognition test with an increased preference to explore the familiar object, more profound reduced body temperatures than NL-G-F/E4 mice, higher cortical insoluble Aβ42 levels, and an increase in cortical TCR-α transcript levels. Thus, while NL-G-F/E4 mice were more susceptible to KUNV-induced cognitive injury and prolonged viral load in the cortex, NL-G-F/E3 mice were more susceptible to KUNV-induced alterations in activity levels, changes in body temperatures, and increases in cortical insoluble Aβ42 levels and cortical TCR-α transcript levels. Some outcome measures were similarly affected in NL-G-F/E3 and NL-G-F/E4 mice. Reduced hippocampus-dependent spontaneous alternation and increased cortical transcript levels of IFN-γ, CXCL10, TCR-α, and TNF-α were seen KUNV-infected NL-G-F/E3 and NL-G-F/E4 mice. Finally, genotype differences in some outcome measures were seen independent of viral exposure. NL-G-F/E4 mice showed higher levels of anxiety, higher cortical soluble Aβ40 levels, and lower cortical soluble and insoluble Aβ42/40 ratio than NL-G-F-E3 mice.

**Table 1:** Summary of effects of KUNV treatment in NL-G-F/E3 and NL-G-F/E4 mice.

Measure	NL-G-F/E3	NL-G-F/E4	Genotype comparison of KUNV effects
Spatial habituation in the open field	KUNV-infected NL-G-F/E3 mice not impaired	KUNV-infected NL-G-F/E4 mice impaired	NL-G-F/E3 > NL-G-F/E4
Measures of anxiety on day 1 of the open field	Comparable anxiety levels in KUNV-infected NL-G-F/E3 mice	Increased anxiety levels in KUNV-infected NL-G-F/E4 mice	NL-G-F/E4 > NL-G-F/E3
Distance moved in the open field containing objects	Lower activity levels in KUNV-infected NL-G-F/E3 mice	Comparable activity levels in PBS-treated and KUNV-infected NL-G-F/E4 mice	NL-G-F/E4 > NL-G-F/E3
Novel object recognition	Neophobia in KUNV-infected NL-G-F/E3 mice	Neophobia in PBS-treated NL-G-F/E4 mice; no preference in KUNV-infected NL-G-F/E4 mice	NL-G-F/E3 = NL-G-F/E4
Spontaneous alternation in the Y maze	Reduced spontaneous alternation in UNV-treated NL-G-F/E3 mice	Reduced spontaneous alternation in KUNV-treated NL-G-F/E4 mice	NL-G-F/E3 = NL-G-F/E4
Circadian body temperatures	Lower body temperatures in KUNV- than PBS-treated NL-G-F/E3 mice	Lower body temperatures in KUNV- than PBS-treated NL-G-F/E4 mice	NL-G-F/E3 > NL-G-F/E4
Insoluble cortical Aβ42 levels	Higher levels in KUNV- than PBS-treated NL-G-F/E3 mice	Comparable levels in KUNV- and PBS-treated NL-G-F/E4 mice	NL-G-F/E3 > NL-G-F/E4
Soluble cortical Aβ40 levels	No effects of KUNV on levels in NL-G-F/E3 mice	No effects of KUNV on levels in NL-G-F/E4 mice	NL-G-F/E3 = NL-G-F/E4
Insoluble cortical Aβ42/40 ratio	No effects of KUNV on ratio in NL-G-F/E3 mice	No effects of KUNV on ratio in NL-G-F/E4 mice	NL-G-F/E3 = NL-G-F/E4
Soluble cortical Aβ42/40 ratio	No effects of KUNV on ratio in NL-G-F/E3 mice	No effects of KUNV on ratio in NL-G-F/E4 mice	NL-G-F/E3 = NL-G-F/E4
Viral load in cortex at 7 weeks post-inoculation			NL-G-F/E4 > NL-G-F/E3 More NL-G-F/E4 than NL-G-F/E3 mice with viral load in cortex
Cortical TCR-α transcript levels	Higher in KUNV-infected NL-G-F/E3 mice without detectable virus than in control NL-G-F/E3 mice	Comparable levels in PBS-treated and KUNV-infected NL-G-F/E3 mice without detectable virus	NL-G-F/E3 > NL-G-F/E4

Seven weeks after viral inoculation, viral loads in the cortex were detected in more KUNV-infected NL-G-F/E4 than NL-G-F/E3 mice. This pattern is consistent with that with other viruses, including HSV-1 (Burgos et al., 2006), herpes simplex virus type-1 and human immunodeficiency virus (Chen et al., 2023) and the association of apoE4 with more severe COVID19 (Ciurleo et al., 2023). Compared to apoE3, apoE4 is a risk factor to develop AD (Raber et al., 2004; Farrer et al., 1997) and cognitive injury following various environmental challenges (Raber, 2004; Liu et al., 2013). (Re) activation of neurotropic viruses might increase the risk of developing AD and other neurodegenerative conditions. Human cytomegalovirus (HCMV)-infected cerebral organoids showed enhanced AD pathology (Aβ42 and pTau-212) and neuronal death (Readhead et al., 2025). The association with AD is proposed for reactivated Herpes Simplex virus (Itzhaki, 1994) and other neurotropic viruses as well (Burt et al., 2008; Siddiqui et al., 2018; Hoshino et al., 2007; Burgos et al., 2006). The viral association is not limited to AD either and associated with other neurodegenerative conditions as well, including Parkinson’s Disease, Multiple Sclerosis, Amyotrophic Lateral Sclerosis, and vascular dementia (Levine et al., 2023). However, insoluble Aβ42 levels in cortex were increased following exposure to the KUNV strain of WNV in NL-G-F/E3, but not in NL-G-F/E4, mice. Consistent with the pattern seen in 6-month-old NL-G-F/E3 and NL-G-F/E4 mice (Holden et al., 2022), the insoluble cortical Aβ42 levels were lower in NL-G-F/E4 than NL-G-F/E3 mice. Therefore, it is possible that because of these baseline differences we might not have seen an increase in insoluble cortical Aβ42 levels in NL-G-F/E4 mice. Regardless, these data suggest that there might not be a simple relationship between viral load and amyloid pathology. In mice showing viral load in cortex 7 weeks following exposure, all four immune measures were elevated in NL-G-F/E4 mice and the NL-G-F/E3 and NL-G-F/E4 mice combined. These data suggest that persistent viral load is associated with chronic neuroinflammation that likely drives the physiological, behavioral, and cognitive alterations seen in the mice. In contrast to the cortex, while the pattern was the same in the hippocampus it did not reach significance. As often seen for other measures, this might simply reflect the relatively smaller hippocampal tissue and the associated increase in variability between values in hippocampal measures of the same group.

Of the four immune measures analyzed 7 weeks after exposure, cortical levels of TCR-α transcripts were higher in NL-G-F/E3 mice following KUNV exposure and clearance of detectable virus in the cortex compared to PBS-treated genotype-matched controls. In PBS-treated mice, cortical levels of TCR-α transcripts were higher in NL-G-F/E4 than NL-G-F/E3 mice. While TCR-α on CD8 + T cells is important for the response to WNV infection and infiltration of these cells into the brain (Kitaura et al., 2011), we cannot exclude that based on baseline differences in cortical levels of TCR-α transcripts we did not see an increase in NL-G-F/E4 mice.

Reduced hippocampus-dependent spontaneous alternation was seen KUNV-infected NL-G-F/E3 and NL-G-F/E4 mice. However, for other cognitive measures, NL-G-F/E4 mice were more susceptible than NL-G-F/E3 mice to detrimental effects of KUNV exposure. KUNV-infected NL-G-F-/E4 mice showed impairments in hippocampus-dependent spatial habituation learning in the open field and did not distinguish exploring the two objects in the novel object recognition test. These results are consistent with the increased susceptibility of those with apoE4 to various environmental challenges (Raber, 2004; Liu et al., 2013) and the cognitive impairments reported in mice expressing apoE4 in neurons (Raber et al., 2000; Raber et al., 1998) or astrocytes (Van Meer et al., 2007) and on an apoE knockout background.

In contrast to increased susceptibility of NL-G-F/E4 mice to develop cognitive injury following viral exposure, NL-G-F/E3 mice were more susceptible to develop behavioral and physiological changes; KUNV-infected NL-G-F/E3 mice showed lower activity levels in the open field containing objects, increased preference to explore the familiar object in the object recognition test, and more profound reduced body temperatures than those seen in KUNV-infected NL-G-F/E4 mice. The increase preference to explore the familiar object (neophobia) is anxiety-related (File, 2001). However, KUNV-infected NL-G-F/E4, but not NL-G-F/E3, mice showed increased measures of anxiety in the open field. The distinction between viral effects on exploring novel objects and time spent in the more anxiety-provoking center of the open field is consistent with the preferential exploring of familiar objects without changes in exploratory behavior seen in mutant mice lacking RICH2 (RhoSAP: RhoGAP synapse-associated protein), a phenotype that relates to developmental disorders such as autism spectrum disorder (Sarowar et al., 2017).

NL-G-F/E4 mice showed higher body temperatures than NL-G-F/E3 mice. These data are consistent with the higher body temperatures in standard chow fed regular E4 than E3 mice (Arbones-Mainar et al., 2017). KUNV-infected NL-G-F/E3 and NL-G-F/E4 mice showed lower body temperatures than genotype-matched PBS-treated mice. These effects were more pronounced in NL-G-F/E3 than NL-G-F/E4 mice. More specifically, during the D9-D16 dark and light periods and during the D33-D38 dark period, body temperatures were lower in KUNV- than PBS-treated NL-G-F/E3 mice. These effects were genotype-dependent and not seen in NL-G-F/E4 mice. Hypothermia can occur after severe viral infection, including following exposure to COVID-19 and Respiratory Syncytial Virus (RSV), is associated with higher mortality than hyperthermia or normothermia in patient with sepsis (Werner et al., 2025), and seen in mice following exposure to influenza virus and related to pulmonary lesions (Yang and Evans, 1961). WNV exposure can cause pulmonary complications due to persistent neuromascular weakness (Bampali et al., 2022; Szeto et al., 2024), and it is conceivable that there might be genotype differences in lung injury following KUNV exposure in our mouse model.

IFN-γ and TNF-α have been reported to induce and modulate hypothermia (Leon, 2004). Due to the nature for testing mice in an ABSL-2 facility, we were not allowed access to the mice the first 4 weeks following viral exposure. Cytokines like IFN-γ and TNF-α might have been elevated more in KUNV-infected NL-G-F/E3 than NL-G-F/E4 mice. A further limitation of this study was that all behavioral testing occurred within a biosafety cabinet, preventing us from using some behavioral and cognitive tests that require more space and cannot be performed within a biosafety cabinet.

In summary, the data of the current study show that exposure to the KUNV strain of WNV affects physiological, behavioral, cognitive, amyloid pathology, viral load, and immune measures in middle-aged NL-G-F mice in an apoE isoform-dependent fashion. Future efforts are warranted to determine whether these effects are age-dependent or are also seen in adult (6-month-old) mice, a time point at which amyloid pathology is seen in NL-G-F (Saito et al., 2014; Holden et al., 2022; Masuda et al., 2016) and NL-G-F/E3 and NL-G-F/E4 mice (Holden et al., 2022) and to include more molecular outcome measures to understand which pathways are affected by KUNV in an apoE isoform-dependent fashion.

## Data Availability

The original contributions presented in the study are included in the article/Supplementary material, further inquiries can be directed to the corresponding author.
