# Supplementary material for: Effects of West Nile virus on behavioral and cognitive performance, cortical Aβ pathology, viral loads, and immune measures of middle-aged NL-G-F/E3 and NL-G-F/E4 mice
Source: Front Aging Neurosci. 2025 Jun 17;17:1600119. doi: 10.3389/fnagi.2025.1600119 (PMC12209204; doi:10.3389/fnagi.2025.1600119)
Supplement: Supplementary file 1 [file Table_1.docx]

**Effects of West Nile Virus on behavioral and cognitive performance, cortical Ab pathology, viral loads, and immune measures of middle-aged NL-G-F/E3 and NL-G-F/E4 mice**

Abigail O’Neil^1^, Christopher J. Parkins^2^, Alexandra Pederson^1^, Elizabeth Saltonstall^1^, Emily Bunnell^1^, Ria Aggarwal^1^, Phoebe Sandholm^1^, Kat Kessler^1^, Henry F Harrison^2^, Jessica L. Smith^2^, Alec J. Hirsch^2^, Jacob Raber*^1,3,4,5^

^1^ Department of Behavioral Neuroscience, Oregon Health & Science University, Portland, Oregon 97239

^2^ Vaccine and Gene Therapy Center, ONPRC, Oregon Health & Science University, Portland, Oregon 97239

^3^ Department of Neurology, Oregon Health & Science University, Portland, Oregon 97239

^4^ Department of Radiation Medicine, Oregon Health & Science University, Portland, Oregon 97239

^5^ Division of Neuroscience, ONPRC, Oregon Health & Science University, Portland, Oregon 97239

*** Correspondence:**Corresponding Author
[raberj@ohsu.edu](mailto:raberj@ohsu.edu); 503 494-1524


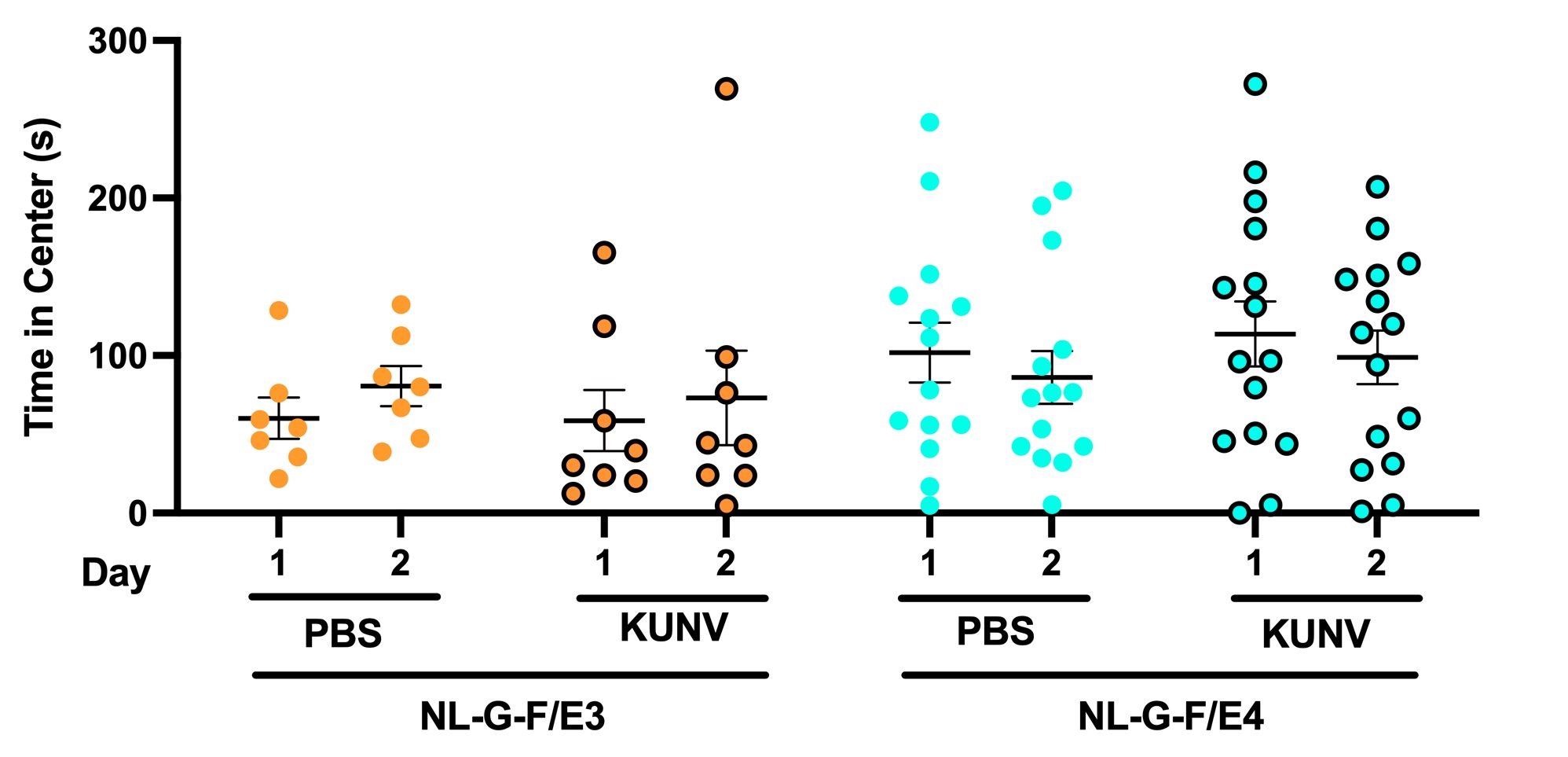


**S1.** Time PBS- and Kunjin-treated NL-G-F/E4 and NL-G-F/E4 mice spent in the center of the open field containing objects. NL-G-F/E3. PBS: *n* = 7; KUNV: *n* = 8; NL-G-F/E4. PBS: *n* = 14; KUNV: *n* = 15.

*Detailed statistical analyses of the circadian body temperatures*

During the D1-D8 dark periods (Figs. 6A, 6B), there was an effect of day (*F*(3.991,143.659) = 5.940, *p <* 0.001) and genotype (*F*(1,36) = 5.858, *p =* 0.021), with higher body temperatures in NL-G-F/E4 than NL-G-F/E3 mice. During the D1-D8 light periods, there was also an effect of day (*F*(4.488,161.563, Greenhouse-Geisser correction) = 5.940, *p <* 0.001) and genotype (*F*(1,36) = 6.583, *p =* 0.015), with higher body temperatures in NL-G-F/E4 than NL-G-F/E3 mice.

In NL-G-F/E3 mice during the D1-D8 dark periods, there was a trend towards an effect of day (*F*(2.995,28.947) = 2.514, *p =* 0.080, Greenhouse-Geisser correction) and a trend towards an effect of treatment (*F*(1,10) = 3.747, *p =* 0.082). In NL-G-F/E4 mice during the D1-D8 dark period, there was an effect of day (*F*(3.848,96.188) = 2.514, *p =* 0.001, Greenhouse-Geisser correction).

In NL-G-F/E3 mice during the D1-D8 light periods, there was an effect of day (*F*(7,70) = 7.171, *p* < 0.001). In NL-G-F/E4 mice during the D1-D8 light periods, there was also an effect of day (*F*(4.449,111.236) = 12.843, *p* < 0.001, Greenhouse-Geisser correction).

During the D9-D16 dark periods, there was an effect of genotype (*F*(1,36) = 6.616, *p =* 0.014), with higher body temperatures in NL-G-F/E4 than NL-G-F/E3 mice (Fig. 6C, 6D). During the D9-D16 light periods, there was also an effect of treatment (*F*(1,36) = 5.870, *p =* 0.021), with higher body temperatures in PBS- than KUNV-infected mice, an effect of genotype (*F*(1,36) = 4.486, *p =* 0.041), with higher body temperatures in NL-G-F-E4 than NL-G-F/E3 mice, and a trend towards a genotype x treatment interaction (*F*(1,36) = 2.882, *p =* 0.098). As one mouse died during the study, we also analyzed the D9-D16 data with the data of that mouse removed. With data of that mouse removed for the dark D9-16 periods, there was an effect of day (*F*(3.598,125.913) = 4.219, *p =* 0.004, Greenhouse-Geisser correction), a day x treatment x genotype interaction (*F*(3.598,125.913) = 4.002, *p =* 0.006, Greenhouse-Geisser correction), with high body temperatures in PBS- than KUNV-infected NL-G-F/E3, but not NL-G-F/E4, mice duringD12-D16), an effect of genotype (*F*(1,35) = 4.971, *p =* 0.032), with higher body temperatures in NL-G-F-E4 than NL-G-F/E3 mice, and a trend towards a day x genotype interaction (*F*(3.598,125.913) = 2.481, *p =* 0.053, Greenhouse-Geisser correction). With data of that mouse removed for the light D9-16 periods, there was an effect of treatment (*F*(1,35) = 4.448, *p =* 0.042), with higher body temperatures in PBS- than KUNV-infected mice.

In NL-G-F/E3 mice during the D9-D16 dark periods, there was an effect of day (*F*(7,70) = 5.499, *p* < 0.001) and a day x treatment interaction (*F*(7,70) = 3.179, *p* = 0.006). While the body temperature was comparable in PBS- and KUNV-infected NL-G-F/E3 mice on D9-D10, the body temperatures in KUNV-infected NL-G-F/E3 mice was increasingly lower than that in PBS-treated NL-G-F/E3 mice over D11-D16. In NL-G-F/E4 mice during the D9-D16 dark periods, there were no significant effects. In NL-G-F/E3 mice during the D9-D16 light periods, there was a day x treatment interaction (*F*(2.508,25.081) = 3.239, *p* = 0.046, Greenhouse-Geisser correction). While the body temperature was comparable in PBS- and KUNV-infected NL-G-F/E3 mice on D9-D10, the body temperatures in KUNV-infected NL-G-F/E3 mice was increasingly lower than that in PBS-treated NL-G-F/E3 mice over D11-D16. There was also a trend towards an effect of treatment (*F*(1,10) = 4.521, *p =* 0.059). In NL-G-F/E4 mice during the D9-D16 light periods, there were no significant effects.

During the D17-24 dark periods, there was an effect of genotype (*F*(1,35) = 4.497, *p =* 0.041) (Figs. 6E, 6F), with higher body temperatures in NL-G-F/E4 than NL-G-F/E3 mice. During the D17-24 light periods, there was an effect of day (*F*(4.471,156.568) = 6.137, *p* < 0.001, Greenhouse-Geisser correction), a day x treatment interaction (*F*(4.471,156.568) = 3.028, *p* = 0.016, Greenhouse-Geisser correction), a day x treatment x genotype interaction (*F*(4.471,156.568) = 2.581, *p* = 0.034, Greenhouse-Geisser correction), and an effect of genotype (*F*(1,35) = 6.113, *p =* 0.018), with higher body temperatures in NL-G-F/E4 than NL-G-F/E3 mice.

When the D17-24 dark periods in NL-G-F/E3 mice and NL-G-F/E4 mice were analyzed separately there were no significant effects in either genotype. In NL-G-F/E3 mice during the D17-D24 light periods, there was an effect of day (*F*(7,70) = 3.516, *p* = 0.003) and a day x treatment interaction (*F*(7,70) = 3.263, *p* = 0.005). While the body temperature was comparable in NL-G-F/E3 mice on D17-D18, the body temperature was higher in PBS- than KUNV-infected NL-G-f/E3 mice on D23-D24. In NL-G-F/E4 mice during the D17-D24 light periods, there was only an effect of day (*F*(4.113,102.827) = 3.859, *p* = 0.005).

During the D25-32 dark periods, there was an effect of day (*F*(3.709,81.598) = 4.319, *p* = 0.004, Greenhouse-Geisser correction), a day x treatment interaction (*F*(3.709,81.598) = 3.707, *p* = 0.010, Greenhouse-Geisser correction), and an effect of genotype (*F*(1,22) = 8.344, *p* = 0.009) (Fig. 6G, 6H), with higher body temperatures in NL-G-F/E4 than NL-G-F/E3 mice. During the D25-32 light periods, there was an effect of day *F*(3.709,81.598) = 9.208, *p* < 0.001, Greenhouse-Geisser correction) and an effect of genotype (*F*(1,22) = 11.147, *p* = 0.003), with higher body temperatures in NL-G-F/E4 than NL-G-F/E3 mice. Body temperature was higher in Kujin- than PBS-treated NL-G-F/E3 mice on D25-D28 but lower on D31-D32.

During the D33-38 dark periods, there was an effect of genotype (*F*(1,35) = 8.675, *p =* 0.006), with higher body temperatures in NL-G-F/E4 than NL-G-F/E3 mice, a trend towards an effect of day (*F*(2.898,101.425) = 2.163, *p* = 0.099, Greenhouse-Geisser correction), a trend towards a day x treatment interaction (*F*(2.898,101.425) = 2.330, *p* = 0.091, Greenhouse-Geisser correction), and a trend towards a day x genotype x treatment interaction (*F*(2.898,101.424) = 6.137, *p* = 0.053, Greenhouse-Geisser correction) (Fig. 5G, 5H). During the D33-38 light periods, there was a light x treatment x genotype interaction (*F*(2.112,73.920) = 6.137, *p* = 0.053, Greenhouse-Geisser correction), an effect of genotype (*F*(1,35) = 9.052, *p =* 0.005), with higher body temperatures in NL-G-F/E4 than NL-G-F/E3 mice, a trend towards an effect of day (*F*(2.112,73.920) = 6.137, *p* = 0.063, Greenhouse-Geisser correction), a trend towards a day x treatment interaction (*F*(2.112,73.920) = 6.137, *p* = 0.053, Greenhouse-Geisser correction), a trend towards a day x genotype interaction (*F*(2.112,73.920) = 6.137, *p* = 0.059, Greenhouse-Geisser correction).

In NL-G-F/E3 mice during the D33-D38 dark periods, there was a day x treatment interaction (*F*(5,50) = 2.413, *p =* 0.049). On D33, D34, D37, D38, but not on D35, body temperature was higher in PBS- than KUNV-infected NL-G-F/E3 mice. In NL-G-F/E4 mice during the D33-D38 dark periods, there were no significant effects. In NL-G-F/E3 and NL-G-F/E4 mice during the D33-D38 light periods, there were no significant effects.
